# Fat Embolization Syndrome in Sickle Cell Disease: A Case Report

**DOI:** 10.7759/cureus.103578

**Published:** 2026-02-14

**Authors:** Narjis AlQassab, Sara S Radhi, Fatema A Alnashaba, Mahmood B Dhaif, Ali M Haider Ali, Abdulrahman Al-Majmuei

**Affiliations:** 1 Accident and Emergency, Salmaniya Medical Complex, Muharaq, BHR; 2 Medicine, Royal College of Surgeons in Ireland, Manama, BHR

**Keywords:** acute chest syndrome (acs), cerebral fat embolism, exchange transfusion, fat embolization syndrome, intensive care unit, sickle cell disease, starfield pattern

## Abstract

Sickle cell disease (SCD) is a common hereditary hemoglobinopathy in Bahrain and is associated with a broad spectrum of acute and chronic multisystem complications. Fat embolization syndrome (FES) is a rare but life-threatening complication of SCD that results from the systemic release of fat globules, typically originating from ischemic bone marrow, leading to pulmonary, neurological, and multiorgan dysfunction. Early diagnosis is often challenging due to its non-specific clinical presentation and overlap with other sickle cell complications.

We report the case of a 22-year-old male with SCD who presented with a vaso-occlusive crisis (VOC) and subsequently developed acute chest syndrome and pulmonary embolism during hospitalization. The patient later experienced acute neurological deterioration, and brain magnetic resonance imaging (MRI) demonstrated the characteristic “starfield pattern,” supporting the diagnosis of cerebral fat embolization. His clinical course was further complicated by acute kidney injury, sepsis-induced cardiomyopathy, and tension pneumothorax, requiring prolonged intensive care management. Treatment included repeated red blood cell exchange transfusions alongside comprehensive supportive care. The patient demonstrated gradual neurological and cardiopulmonary recovery following an extended intensive care unit stay.

This case highlights the diagnostic complexity of FES in SCD and emphasizes the importance of early clinical suspicion in patients presenting with respiratory failure or neurological deterioration following VOC. MRI, particularly the identification of the starfield pattern, serves as a key diagnostic tool. Prompt recognition and initiation of exchange transfusion remain critical in improving clinical outcomes and reducing mortality.

## Introduction

Sickle cell disease (SCD) is one of the most common hereditary hematological disorders in Bahrain, with an estimated overall prevalence of approximately 1% of the population [1,2]. National newborn screening programs report that approximately 2% of screened neonates are diagnosed with SCD, while nearly 18% of the population carries the sickle cell trait [1,2]. Fat embolization syndrome (FES) is a rare but potentially life-threatening complication of SCD, defined as a clinical condition resulting from systemic embolization of fat droplets, typically originating from bone marrow, leading to respiratory, neurological, and multisystem dysfunction.

Fat embolism is classically associated with long bone fractures due to the release of fat droplets from disrupted bone marrow into the bloodstream [3]. In SCD, the presumed pathogenesis typically occurs 12-72 hours following a vaso-occlusive crisis (VOC), during which ischemic bone marrow necrosis results in the release of marrow fat globules into the systemic circulation [3]. These fat globules may embolize to the lungs, resulting in pulmonary embolism (PE), acute respiratory distress syndrome (ARDS), and respiratory failure, or to the brain, leading to neurological manifestations such as altered mental status or seizures [3]. The resulting microvascular obstruction may lead to multiorgan dysfunction and death. Additionally, FES in SCD may trigger a systemic inflammatory response and a coagulopathic cascade [4,5].

FES remains an underrecognized complication due to the non-specific nature of its early clinical presentation, which may overlap with other conditions such as severe sepsis, acute chest syndrome (ACS), or hypoxic-ischemic cerebral injury. Diagnosis is primarily clinical and is often guided by established diagnostic frameworks such as Gurd’s criteria, supported by radiological findings and laboratory abnormalities [6]. A characteristic neuroimaging finding associated with cerebral fat embolization is the “starfield pattern” observed on diffusion-weighted magnetic resonance imaging (MRI), representing diffuse microinfarctions and embolic phenomena [7]. Early recognition of FES is critical, as timely red blood cell exchange transfusion has been associated with significant reductions in morbidity and mortality [8].

## Case presentation

A 22-year-old male with known SCD and recurrent hospitalizations for VOC presented to the emergency department on 20 January 2025 with generalized body pain. He was diagnosed with a simple VOC and admitted for analgesia and intravenous hydration. Initial laboratory evaluation showed a white blood cell (WBC) count of 21.08×10⁹/L, hemoglobin (Hb) of 10.10 g/dL, and a platelet count of 344×10⁹/L.

On day two, he developed acute dyspnea, oxygen desaturation, and tachypnea. Arterial blood gas (ABG) analysis revealed a pH of 7.41, partial pressure of carbon dioxide or PaCO₂ of 32.7 mmHg, and partial pressure of oxygen or PaO₂ of 110 mmHg while receiving oxygen via a non-rebreather mask at 15 L/min, consistent with compensated respiratory alkalosis in the setting of acute respiratory compromise. Serum sodium was 130 mmol/L, potassium was 4.2 mmol/L, and bicarbonate was 22 mmol/L.

Urgent computed tomography pulmonary angiography demonstrated a right subsegmental PE with bilateral ground-glass opacities and patchy consolidation. Concurrent laboratory studies showed worsening leukocytosis, with WBC rising from 21.08×10⁹/L to 26.1×10⁹/L. Hb decreased from 10.10 g/dL to 7.1 g/dL, and platelets declined from 344×10⁹/L to 100×10⁹/L. Reticulocyte count was elevated at 242.8×10⁹/L (7.1%). These findings were consistent with ACS in the context of SCD.

The progressive anemia and thrombocytopenia, together with rising leukocytosis and hyperbilirubinemia, suggested ongoing hemolysis and a systemic inflammatory response. These laboratory trends were concerning for severe VOC with associated bone marrow necrosis, a recognized precipitant of fat embolization in SCD. The elevated WBC and inflammatory markers also raised suspicion for superimposed infection and sepsis, both of which remained in the differential diagnosis during clinical deterioration.

At the onset of respiratory decline, vital signs showed a heart rate of 112 beats per minute, blood pressure of 135/86 mmHg, and oxygen saturation of 93% on supplemental oxygen. He was started on an unfractionated heparin infusion and broad-spectrum antibiotics covering typical and atypical organisms. Within 24 hours, oxygen requirements escalated to 60 L/min via high-flow nasal cannula, prompting transfer to the intensive care unit (ICU).

On ICU admission, Hb remained 7.1 g/dL, platelets 100×10⁹/L, and WBC 26.1×10⁹/L. Liver function testing revealed hyperbilirubinemia, with total bilirubin of 115 µmol/L and direct bilirubin of 16 µmol/L, supporting ongoing hemolysis. Complete laboratory data are summarized in Table 1.

**Table 1:** Summary of laboratory investigations during admission and clinical deterioration

Parameter	Result	Unit	Reference range
Hemoglobin	10.10 → 7.1	g/dL	13.5-17.5
White Blood Cell Count	21.08 → 26.1	×10⁹/L	4-11
Platelet Count	344 → 100	×10⁹/L	150-400
Reticulocyte Count (Absolute)	242.8	×10⁹/L	25-75
Reticulocyte Percentage	7.1	%	0.5-2.5
Urea	3.5	mmol/L	2.5-7.8
Creatinine (Baseline)	56	µmol/L	60-110
Total Bilirubin	115	µmol/L	3-21
Direct Bilirubin	16	µmol/L	0-7
Albumin	40	g/L	35-50
Total Protein	67	g/L	60-80
Sodium	130	mmol/L	135-145
Potassium	4.2	mmol/L	3.5-5.0
Bicarbonate	22	mmol/L	22-26
Partial Pressure of Oxygen (PaO₂)	110	mmHg	80-100
Partial Pressure of Carbon Dioxide (PaCO₂)	32.7	mmHg	35-45
Troponin I	1.300	ng/mL	<0.05

**Figure 1:**
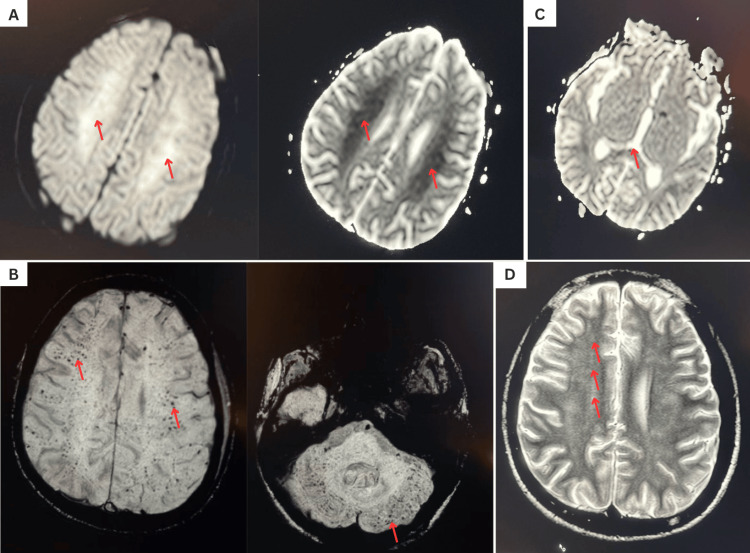
Magnetic resonance imaging findings consistent with cerebral fat embolization syndrome (A) Diffusion-weighted imaging demonstrating diffusion restriction within the bilateral corona radiata. (B) Susceptibility-weighted imaging showing multiple punctate microhemorrhages involving the cerebral white matter, subcortical regions, and cerebellum. (C) Diffusion restriction involving the splenium of the corpus callosum, producing a characteristic starfield pattern. (D) T2 fluid-attenuated inversion recovery imaging demonstrating ill-defined patchy white matter hyperintensities in the bilateral corona radiata.

The patient subsequently developed acute confusion with reduced alertness and disorientation to time, place, and person, with a Glasgow Coma Scale score of 10/15 [9]. A non-contrast CT brain showed no acute abnormalities, and an MRI of the brain was obtained. T2 fluid-attenuated inversion recovery sequences demonstrated diffuse hyperintensities in the bilateral centrum semiovale and splenium of the corpus callosum. Diffusion-weighted imaging revealed widespread diffusion restriction with punctate microhemorrhages, producing the characteristic “starfield pattern.” This pattern represents numerous scattered foci of restricted diffusion corresponding to microinfarctions caused by embolized fat droplets within the cerebral microvasculature and is considered highly suggestive of cerebral fat embolization [7]. Corresponding MRI findings are presented in Figure 1.

Multiple patchy bone marrow infarctions were suspected secondary to VOC and ischemic marrow necrosis, which are recognized sources of fat embolization in SCD. The patient was intubated shortly after ICU admission because of severe type I respiratory failure, and vasopressors were initiated to support hemodynamic stability. Baseline renal function showed urea of 3.5 mmol/L and creatinine of 56 µmol/L. During the ICU course, renal function deteriorated, with a significant rise in creatinine consistent with acute kidney injury, likely multifactorial in etiology, including fat embolism, nephrotoxic antibiotics, and sepsis.

As part of the evaluation for multiorgan dysfunction, cardiac biomarkers demonstrated elevated troponin I of 1.300 ng/mL (reference range: <0.05 ng/mL). This prompted bedside transthoracic echocardiography (TTE), which revealed global hypokinesia, biventricular dysfunction, and an ejection fraction of 40%. These findings were consistent with sepsis-induced cardiomyopathy, defined as a transient and reversible myocardial dysfunction occurring in patients with sepsis without structural or regional heart disease [10].

During mechanical ventilation, the patient developed acute oxygen desaturation, hypotension, and tachycardia. Chest radiography demonstrated a large pneumothorax, likely secondary to positive-pressure ventilation and barotrauma. In the presence of hemodynamic compromise, this met the diagnostic criteria for tension pneumothorax.

Management required a multidisciplinary approach involving hematology, cardiology, respiratory medicine, neurology, and critical care teams. The patient developed multiple severe complications of SCD, resulting in a prolonged ICU course. Early in the admission, he experienced two major respiratory complications. The first was a right subsegmental PE, which was managed with unfractionated heparin infusion for five days, followed by transition to a direct oral anticoagulant (apixaban). Concurrently, imaging and laboratory findings suggested bilateral lower lobe pneumonia, supported by elevated inflammatory markers, neutrophilic leukocytosis, and procalcitonin levels. Progressive respiratory deterioration required escalation of antimicrobial therapy and intubation within 72 hours of admission.

Neurological deterioration occurred as oxygen requirements increased. CT brain failed to identify an acute structural cause, whereas MRI brain demonstrated findings consistent with FES in the clinical context of SCD.

There is no definitive treatment for FES. Management focuses on supportive care, respiratory stabilization, red cell exchange transfusion, and neurological monitoring [7]. Additional supportive measures include serial ABG monitoring, surveillance of renal and hepatic function, coagulation assessment, and monitoring for evolving organ failure [7]. In this case, mechanical ventilation was required because of severe hypoxemia, altered mental status, and an ARDS-like radiographic pattern associated with severe ACS and sepsis.

The patient underwent 10 exchange transfusions during the ICU stay to maintain HbS levels below the therapeutic threshold of 35%. Exchange transfusion improves oxygen delivery, enhances tissue perfusion, and reduces circulating sickled erythrocytes, thereby limiting further fat embolization and improving neurological outcomes through maintenance of normotension, normoxia, normocapnia, and normoglycemia [7]. Hemodynamic stability was supported with intravenous fluids and vasopressors to maintain a mean arterial pressure above 65 mmHg.

Neurology recommended seizure prophylaxis with levetiracetam. The patient’s acute kidney injury was likely multifactorial, including renal fat embolization, nephrotoxic antibiotic exposure (meropenem and vancomycin), and sepsis-associated hypoperfusion.

After 14 days, respiratory function improved significantly, with follow-up chest radiographs demonstrating resolution of prior consolidative changes. Cardiovascular function also improved, allowing gradual discontinuation of vasopressors. Serial cardiac biomarkers decreased, and repeat TTE demonstrated normalization of ejection fraction to 60% without residual ventricular dysfunction.

Neurological recovery progressed more slowly, largely due to prolonged mechanical ventilation and ICU-related complications. The patient developed hospital-acquired infections, including ventilator-associated pneumonia and catheter-associated urinary tract infection, which extended the ICU stay to seven weeks. Despite these complications, the patient was successfully extubated after three weeks of ICU admission, followed by gradual neurological improvement. Intensive physiotherapy was required because of diffuse symmetric weakness.

Management of tension pneumothorax involved emergency needle decompression followed by definitive chest tube placement. The thoracic surgery team monitored the patient, and the chest tube was removed after radiographic confirmation of lung re-expansion and absence of persistent air leak.

## Discussion

This case illustrates FES in a young patient with SCD who initially presented with a seemingly uncomplicated VOC but subsequently developed multiple severe complications. The clinical course progressed from PE and ACS to sepsis, ARDS-like respiratory failure, and acute neurological deterioration, with neuroimaging ultimately supporting the diagnosis of FES. The patient required prolonged ICU admission complicated by hospital-acquired infections and treatment-related adverse events, followed by extensive inpatient and outpatient rehabilitation and multidisciplinary follow-up.

FES in SCD remains an underrecognized and underdiagnosed complication because of its non-specific clinical presentation and overlap with other acute SCD-related syndromes, particularly severe sepsis and ACS. Classically, FES presents with a triad of respiratory distress, neurological impairment, and petechial rash. Respiratory manifestations typically include hypoxemia, tachypnea, and ARDS-like radiologic findings, while neurological symptoms range from confusion to seizures and coma. Petechial rash is less frequently observed in FES associated with SCD. The presence of respiratory deterioration and altered mental status following VOC should raise clinical suspicion for FES [11].

Although the starfield pattern on MRI is highly suggestive of cerebral fat embolization, it is not pathognomonic [4]. Similar diffusion restriction patterns may be observed in cytotoxic lesions of the corpus callosum, hypoxic-ischemic injury, severe infection, and metabolic disturbances [12]. In the present case, these alternative diagnoses were carefully considered. However, the close temporal relationship with VOC, rapid respiratory decline, characteristic laboratory abnormalities indicating hemolysis and systemic inflammation, and the presence of multiorgan involvement collectively supported FES as the most likely diagnosis.

Multiorgan dysfunction is a common feature of FES and may manifest as hemodynamic instability, laboratory evidence of organ injury such as elevated liver enzymes or renal impairment, and features suggestive of bone marrow failure [13]. Management is primarily supportive and begins with early resuscitation and aggressive respiratory support, including ARDS-directed ventilatory strategies tailored to individual patient requirements [13]. Patients frequently require ICU-level monitoring and intervention. Neurological management includes seizure treatment or prophylaxis and close neuro-observation [14].

Red blood cell exchange transfusion remains a cornerstone of therapy in SCD-associated FES. Exchange transfusion reduces circulating sickled erythrocytes, improves oxygen delivery, enhances tissue perfusion, and limits further fat embolization [11]. Evidence supporting this approach is demonstrated in a systematic review evaluating FES in SCD, which reported substantially lower mortality among patients treated with exchange transfusion (23%) compared with those receiving simple transfusion (59%) or no transfusion (92%) [6]. Concurrent SCD-related complications must also be actively identified and treated. Early recognition of FES and prompt initiation of exchange transfusion are critical determinants of survival.

## Conclusions

FES is a rare but well-recognized and frequently underdiagnosed complication of SCD that primarily results from extensive bone marrow necrosis and subsequent fat globule embolization. This case highlights the importance of maintaining a high index of suspicion for FES in patients with SCD who develop acute respiratory failure or neurological deterioration following VOC. The presence of the characteristic starfield pattern on MRI, when interpreted within the appropriate clinical context, can provide critical diagnostic support. Management remains largely supportive and focuses on organ protection alongside early red blood cell exchange transfusion, which has been shown to significantly reduce morbidity and mortality. Early recognition and timely intervention are essential to improving clinical outcomes in patients with SCD complicated by FES.

## References

[1] El-Hazmi MA, Al-Hazmi AM, Warsy AS (2011). Sickle cell disease in Middle East Arab countries. Indian J Med Res.

[2] Elshaikh RH, Babker AM, Hussein SE, Ahmed KA, Sah AK, Alfeel AH (2026). Pharmacogenomics and opioid efficacy in sickle cell disease. Medicina (Kaunas).

[3] Mossa-Basha M, Izbudak I, Gurda GT, Aygun N (2012). Cerebral fat embolism syndrome in sickle cell anaemia/β-thalassemia: importance of susceptibility-weighted MRI. Clin Radiol.

[4] Kang JH, Hargett CW, Sevilis T, Luedke M (2018). Sickle cell disease, fat embolism syndrome, and "starfield" pattern on MRI. Neurol Clin Pract.

[5] Salam A, Sameer R, Eiman AA (2025). Clinical, laboratory, radiological features, and outcome of acute fat embolism syndrome in sickle cell disease. Sci Rep.

[6] Tsitsikas DA, Vize J, Abukar J (2020). Fat embolism syndrome in sickle cell disease. J Clin Med.

[7] Mohamed EA, Elgari MM, Babker AM, Waggiallah HA (2020). Comparative study of hypercoagulability change in steady state and during vaso-occlusive crisis among Sudanese patients living with sickle cell disease. Afr Health Sci.

[8] Alsharari E, Al Enazi A, Hanafy E, Mustafa M, Abufara F, Altoonisi MM (2025). Indications for blood transfusion and exchange transfusion in sickle cell disease: a single center experience. Cureus.

[9] Teasdale G, Jennett B (1974). Assessment of coma and impaired consciousness. The Lancet.

[10] Boissier F, Aissaoui N (2022). Septic cardiomyopathy: diagnosis and management. J Intensive Med.

[11] Greaves P, Mathew V, Peters C, Rowe S, Amos RJ, Tsitsikas DA (2017). Successful outcome of three patients with sickle-cell disease and fat embolism syndrome treated with intensive exchange transfusion. Clin Case Rep.

[12] Starkey J, Kobayashi N, Numaguchi Y, Moritani T (2017). Cytotoxic lesions of the corpus callosum that show restricted diffusion: mechanisms, causes, and manifestations. Radiographics.

[13] Olivera Arencibia Y, Vo M, Kinaga J, Uribe J, Velasquez G, Madruga M, Carlan SJ (2018). Fat embolism and nonconvulsive status epilepticus. Case Rep Neurol Med.

[14] Han H, Hensch L, Tubman VN (2021). Indications for transfusion in the management of sickle cell disease. Hematology Am Soc Hematol Educ Program.

